# Special Issue “Biomedical Applications of Mesenchymal Stem Cells”

**DOI:** 10.3390/ijms27146158

**Published:** 2026-07-10

**Authors:** Giuliana Mannino, Debora Lo Furno

**Affiliations:** 1 Department of Medicine and Surgery, University of Enna “Kore”, 94100 Enna, Italy; 2 Department of Biomedical and Biotechnological Sciences (BIOMETEC), University of Catania, Via Santa Sofia 97, 95123 Catania, Italy; lofurno@unict.it

## 1. Introduction

Mesenchymal stem/stromal cells (MSCs) have attracted considerable scientific interest over the last three decades due to their broad therapeutic potential in regenerative medicine, tissue engineering, and immune-mediated disorders. Initially, MSC-based therapies were prompted by the ability of these cells to differentiate into mesodermal lineages and contribute directly to cell replacement. However, accumulating evidence has progressively transformed this view. Current knowledge suggests that the therapeutic activity of MSCs is mainly based on their ability to regulate tissue homeostasis through complex interactions with the local microenvironment, involving immunomodulatory, anti-inflammatory, anti-apoptotic, angiogenic, and trophic mechanisms [1,2,3,4,5,6,7,8,9].

Other than acting merely as progenitor cells, MSCs are increasingly recognized as dynamic regulators of biological processes, capable of orchestrating tissue repair through the secretion of cytokines, growth factors, extracellular vesicles (EVs), and other bioactive mediators [4,7,10,11,12,13]. Such activities enable MSCs to influence resident cells, modulate immune responses, attenuate oxidative stress, and promote regenerative pathways in damaged tissues. Consequently, MSC-based interventions are currently being investigated in a wide range of pathological conditions, including inflammatory, autoimmune, degenerative, and age-related diseases [2,3,5,8].

The Special Issue Biomedical Applications of Mesenchymal Stem Cells was conceived to provide an updated overview of the molecular and cellular mechanisms underlying MSC-mediated tissue repair and immunoregulation. The final collection comprises eight articles authored by 63 researchers from eight countries—China, Italy, Brazil, the United States, Spain, Poland, Serbia, and Switzerland—reflecting the worldwide expansion of MSC research and its relevance across multiple areas of regenerative and translational medicine. The issue includes five original research articles and three review papers, collectively addressing fundamental biological mechanisms, innovative therapeutic approaches, and clinically relevant applications [contributions 1–8].

Although the diseases and experimental models investigated are highly heterogeneous, the contributions converge on several key themes that characterize the current evolution of MSC research. A fundamental observation emerging from this collection is the growing acknowledgement that inflammation and immune dysregulation represent common therapeutic targets across multiple pathological contexts.

This concept is particularly evident in the study investigating adipose-derived MSCs in acute liver injury, where transplantation significantly attenuated tissue damage through suppression of the TLR4/MyD88/NF-κB signaling axis, resulting in reduced inflammatory cytokine production, enhanced antioxidant defenses, and improved hepatic regeneration [contribution 1]. Interestingly, a mechanistically related scenario emerged in the study evaluating the adipose-derived MSC secretome in osteoarthritis [contribution 2]. Here, therapeutic effects were associated with inhibition of NF-κB signaling, suppression of catabolic mediators, preservation of extracellular matrix integrity, and promotion of chondrogenic differentiation.

The convergence of these findings is noteworthy because it suggests that MSCs may exert beneficial effects through the modulation of preserved inflammatory pathways beyond organ-specific pathology. This observation supports the emerging view that successful MSC-based therapies are mainly dependent on the restoration of physiological signaling networks disrupted during disease progression rather than on direct tissue replacement [5,6,8].

The importance of paracrine communication represents another major theme connecting several contributions in this Special Issue. Increasing evidence indicates that many therapeutic effects attributed to MSCs are mediated through secreted bioactive molecules rather than long-term cellular engraftment [4,7,12,13]. In agreement with this hypothesis, the osteoarthritis study demonstrates that the MSC-derived secretome alone can reproduce anti-inflammatory, anti-catabolic, and regenerative effects in inflamed chondrocytes [contribution 2]. Similarly, the review articles addressing corneal regeneration and primary Sjögren’s syndrome highlight the critical role of MSC-derived trophic factors, cytokines, and extracellular vesicles in promoting tissue repair and immune regulation [contributions 3,4].

Overall, these findings reinforce the ongoing transition toward cell-free treatments. Such approaches may offer several practical advantages, including improved safety profiles, easier storage and distribution, reduced manufacturing complexity, and greater opportunities for standardization [7,12]. Nevertheless, important challenges remain. The composition of MSC secretomes is influenced by donor characteristics, tissue origin, culture conditions, and environmental stimuli, generating significant biological variability [7,14]. Therefore, future studies must focus on establishing robust quality-control parameters and potency assays capable of ensuring reproducibility across therapeutic products [15,16].

Another important aspect emerging from this collection concerns the regulation of MSC function by environmental and experimental factors. The study investigating cannabidiol-rich extract priming of equine adipose-derived MSCs demonstrated that pharmacological preconditioning can modulate cytokine expression patterns without compromising cellular viability [contribution 5]. Likewise, the comparative analysis of canine and human Wharton’s jelly-derived MSCs revealed that inflammatory stimulation enhanced immunosuppressive functions in both species, despite species-specific differences in molecular responses [contribution 6] [6]. Such observations are particularly relevant in light of the variability frequently reported in clinical studies. Also in this case, differences in donor age, tissue source and culture procedures remain among the principal obstacles limiting the reproducibility of MSC-based therapies [3,8,15]. Rational strategies aimed at controlling MSC activation and functional commitment may therefore represent an important step toward more predictable clinical outcomes [17].

The integration of MSC biology with biomaterials science constitutes another area of significant innovation represented in this Special Issue. The study employing hydroxyapatite scaffolds functionalized with an osteonectin-mimetic peptide demonstrated enhanced osteogenic differentiation both in vitro and in vivo [contribution 7]. These findings illustrate the growing shift from passive biomaterial scaffolds toward bio-inductive tools capable of actively directing stem cell behavior. Such approaches are likely to play an increasingly important role in regenerative medicine, particularly for the treatment of critical-sized bone defects and complex tissue injuries [17].

Beyond regenerative applications, the collection also provides insights into fundamental disease mechanisms. The study investigating vascular mesenchymal stromal cells and cellular senescence in abdominal aortic aneurysm samples suggests a potential interaction between stromal cell dysfunction, chronic inflammation, and vascular degeneration [contribution 8]. Although preliminary, these observations contribute to the growing body of evidence linking cellular senescence to age-related diseases and raise important questions regarding the role of MSC-related pathways in pathological tissue remodeling [18].

The review articles further expand the translational perspective of the Special Issue. The review on corneal regeneration highlights the potential of MSCs to address the persistent shortage of cornea donors while providing innovative therapeutic options for diseases affecting different corneal layers [contribution 3]. Similarly, the review on severe primary Sjögren’s syndrome provides a comprehensive overview of the mechanisms through which MSCs suppress autoreactive immune responses, promote tissue repair, and potentially offer a targeted therapeutic strategy for complex autoimmune disorders [contribution 4]. Together, these reviews emphasize the broad applicability of MSC-based interventions across various clinical settings.

## 2. New Directions

Future investigations will likely focus on precision regenerative medicine approaches based on a deeper knowledge of MSC biology. Key areas of interest include advanced priming strategies, engineering of extracellular vesicles, development of bioactive scaffolds, and integration of multi-omics technologies to identify molecular signatures associated with therapeutic efficacy [11,12,13].

Single-cell transcriptomics, spatial biology, systems medicine, and artificial intelligence-based analyses are expected to provide innovative insights into MSC heterogeneity and functional specialization. In parallel, comparative studies including veterinary and human models may accelerate translational development and improve the predictive value of preclinical research.

Particular attention should also be devoted to understand how aging, senescence, chronic inflammation, and metabolic alterations affect MSC physiology, given the increasing relevance of these factors in regenerative medicine and age-related diseases [contribution 8] [18].

## 3. Conclusions

The contributions collected in this Special Issue provide a comprehensive and up-to-date overview of mesenchymal stem cell research, highlighting both the progress achieved and the challenges still remaining. Other than demonstrating the broad applicability of MSCs across multiple pathological conditions, these studies together support a paradigm shift in which MSCs are increasingly acknowledged as dynamic regulators of inflammation, tissue homeostasis, and intercellular communication rather than simple replacement cells [1,2,5].
